# Poster Session I - A148 ELECTRONIC CONSULTATION AND REFERRAL SERVICE IS SAFE AND REDUCES THE NEED FOR TERTIARY EVALUATIONS FOR DISORDERS OF GUT-BRAIN INTERACTION IN CHILDREN

**DOI:** 10.1093/jcag/gwaf042.148

**Published:** 2026-02-13

**Authors:** B Chen, M W Carroll, D M Isaac, A S Hudson, K Wong, J A Silverman, H Huynh, P Kawada, J Turner

**Affiliations:** Pediatric gastroenterology, Stollery Children’s Hospital, Edmonton, AB, Canada; Pediatric gastroenterology, Stollery Children’s Hospital, Edmonton, AB, Canada; Pediatric gastroenterology, Stollery Children’s Hospital, Edmonton, AB, Canada; Pediatric gastroenterology, Stollery Children’s Hospital, Edmonton, AB, Canada; Pediatric gastroenterology, Stollery Children’s Hospital, Edmonton, AB, Canada; Pediatric gastroenterology, Stollery Children’s Hospital, Edmonton, AB, Canada; Pediatric gastroenterology, Stollery Children’s Hospital, Edmonton, AB, Canada; Pediatric gastroenterology, Stollery Children’s Hospital, Edmonton, AB, Canada; Pediatric gastroenterology, Stollery Children’s Hospital, Edmonton, AB, Canada

## Abstract

**Background:**

Electronic consultation systems are increasingly used to improve access to specialists in face of growing demand and prolonged wait times. Our Pediatric Gastroenterology (GI) Division in Edmonton launched an eReferral system in 2020 that allows health care providers to seek patient-related advice and make direct referral to our subspecialty.

**Aims:**

Our aim was to review patients sent to e-Referral electronic consultation portal to Pediatric GI and understand their outcomes, including emergency department (ED) visits and/or hospitalizations, and the ultimate need for formal GI consultation and/or endoscopy.

**Methods:**

We undertook a retrospective chart review for eReferral requests to our service from 2022-2023. This timeframe was chosen as it followed the COVID pandemic and allowed for early adoption and local provider familiarity with the eReferral system after introduction in 2020. The study was reviewed and approved by our Health Ethics Board. All eReferrals in the electronic database during this time period were reviewed for the primary reason for referral and time from eReferral to response. Additional information for each patient was obtained from the electronic health care record (ED visit(s) or hospitalization(s) deemed related to the primary reason for eReferral, GI assessment (including endoscopy) and if so, their final GI diagnosis).

**Results:**

A total of 318 patients were referred through e-Referral during the two-year time period. Abdominal pain (38%) and constipation (16%) were the most common reasons for referral. Most patients (83%) were initially referred to our service conventionally, via fax or electronically through the electronic medical record referral module and then re-directed by the triaging physician to seek eReferral advice. The remainder were self-initiated e-Referral requests. 11% of eReferral patients visited the ED for the same GI concern after the eReferral advice request. Three patients required hospital admission, two with constipation for clean-outs and one for dysphagia that was subsequently determined to have a non-GI cause. Only 24% of the eReferral patients were ultimately seen by pediatric GI at a median of 15 weeks from the time of initial eReferral request and 35% of these patients underwent endoscopy. More than half (57%) of the patients seen by pediatric GI were diagnosed with a disorder of gut-brain interaction.

**Conclusions:**

The eReferral portal system appears to be an effective tool in the management and care of pediatric patients with suspected DGBIs referred to our pediatric GI service. Most of these patients have disorders that do not require endoscopy or formal GI consultation. Timely e-advice to referring physicians can expedite enhanced care in the medical home.

**Funding Agencies:**

None

